# ER-Mitochondria Crosstalk during Cerebral Ischemia: Molecular Chaperones and ER-Mitochondrial Calcium Transfer

**DOI:** 10.1155/2012/493934

**Published:** 2012-04-02

**Authors:** Yi-Bing Ouyang, Rona G. Giffard

**Affiliations:** Department of Anesthesia, Stanford University School of Medicine, Stanford, CA 94305-5117, USA

## Abstract

It is commonly believed that sustained elevations in the mitochondrial matrix Ca^2+^ concentration are a major feature of the intracellular cascade of lethal events during cerebral ischemia. The physical association between the endoplasmic reticulum (ER) and mitochondria, known as the mitochondria-associated ER membrane (MAM), enables highly efficient transmission of Ca^2+^ from the ER to mitochondria under both physiological and pathological conditions. Molecular chaperones are well known for their protective effects during cerebral ischemia. It has been demonstrated recently that many molecular chaperones coexist with MAM and regulate the MAM and thus Ca^2+^ concentration inside mitochondria. Here, we review recent research on cerebral ischemia and MAM, with a focus on molecular chaperones and ER-mitochondrial calcium transfer.

## 1. Introduction

Stroke is one of the leading causes of death worldwide and a major cause of long-term disability [1]. Although many clinical trials have been completed in stroke patients, none of these have demonstrated protective efficacy except for thrombolysis [2, 3]. Suggested reasons for this failure include the complex interplay among multiple pathways (for review see [4–6]) including excitotoxicity, mitochondrial dysfunction, acidotoxicity, ionic imbalance, oxidative stress, and inflammation, which can all lead to cell death and irreversible tissue injury.

**Figure 1:**
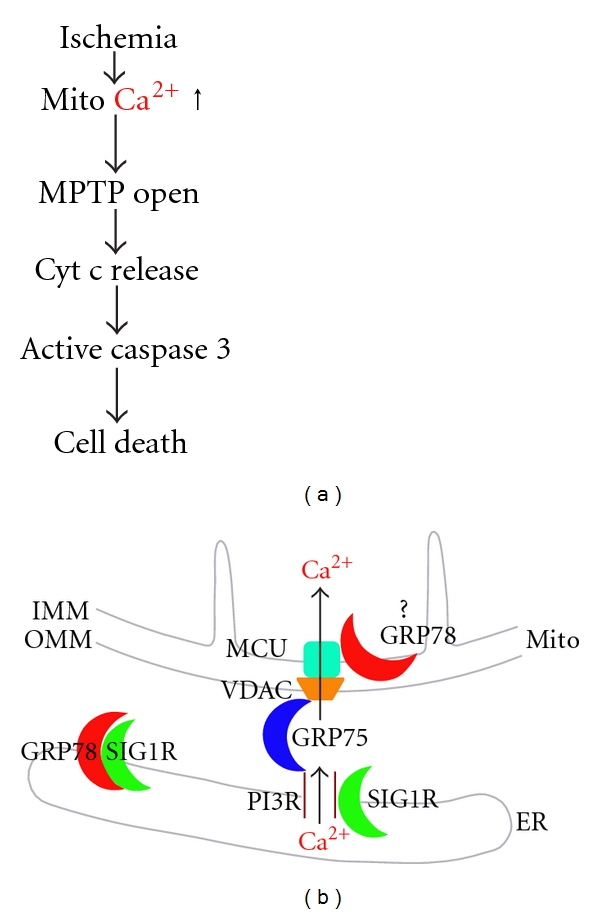
(a) Diagram of cerebral ischemia-induced cell death signaling cascade. (b) Chaperone machinery controls ER-mitochondria Ca^2+^ crosstalk at the MAM. Under normal, resting conditions, SIG1R chaperone forms a complex with GRP78 at the ER. Under stress such as ischemia, SIG1R dissociates from GRP78 and associates with IP3R3 at the MAM, and GRP78 translocates from ER to IMM. ER-mitochondria Ca^2+^ transfer controls cell survival or death decision. Cyt c: cytochrome c; ER: endoplasmic reticulum; GRP75: glucose-regulated protein 75; GRP78: glucose-regulated protein 78 kDa; IMM: inner mitochondrial membrane; IP3R: inositol trisphosphate receptor; MCU: mitochondrial Ca^2+^ uniporter; Mito: mitochondria; MPTP: mitochondrial permeability transition pore; OMM: outer mitochondrial membrane; SIG1R: sigma-1 receptor; VDAC: voltage-dependent anion channel.

A generally accepted cell death pathway after cerebral ischemia is mitochondrial permeability transition (MPT) pore opening (Figure 1(a)). Ischemia leads to energy deprivation and loss of ion homeostasis. As the cells are unable to maintain a negative membrane potential, they depolarize, leading to the opening of voltage-gated calcium channels and release of excitatory amino acids into the extracellular space [7]. This cascade of events leads to a massive entry of calcium and this increase in free cytosolic calcium is transmitted to the matrix of mitochondria by Ca^2+^ channels and exchangers located on the inner mitochondrial membrane. Recently ER stress was found to be one of the effects of excitotoxicity, that is, exposure to toxic levels of excitatory neurotransmitters, with release of Ca^2+^ from the ER via both ryanodine receptors and IP3R, with release from inositol trisphosphate receptors (IP3Rs) leading to mitochondrial Ca^2+^ overload and activation of apoptosis [8]. Excessive increases in matrix Ca^2+^ alter the permeability of mitochondria and finally open the MPT pore [9], causing the release of cytochrome c [10] and other proapoptotic factors into the cytoplasm. The released cytochrome c activates caspase-3, one of the executioner caspases to initiate cell death. Excessive accumulation of calcium in mitochondria is a key factor in the final outcome of the cascade leading to neural cell death (Figure 1(a)) [11].

Mitochondria can accumulate large amounts of calcium through a Ca^2+^-selective channel known as the mitochondrial Ca^2+^ uniporter (MCU) [12, 13]. However, MCU has a relatively low Ca^2+^ affinity [14]. It is interesting that in response to cytosolic Ca^2+^ transients not exceeding concentrations of 1–3 *μ*M, mitochondrial Ca^2+^ concentrations rise almost simultaneously to values above 10 *μ*M [15]. The existence of close contact points between the ER and mitochondria (the mitochondria-associated ER membrane, MAM) is thought to provide a selective direct pathway for calcium from the ER to mitochondria. Upon cell stimulation, the release of high concentrations of Ca^2+^ at MAM leads to the formation of microdomains of high Ca^2+^ concentration that is crucial for efficient Ca^2+^ uptake by mitochondria [16, 17].

Molecular chaperones are a functionally related group of proteins that assist protein folding in cells and protect cells from injury after cerebral ischemia or other stress. It has been demonstrated recently that MAM coexists with many molecular chaperones [18]. The relationship between molecular chaperones and ER-mitochondrial calcium transfer after cerebral ischemia is an emerging research area and is the focus of this mini review. 

## 2. Cerebral Ischemic Models

Animal models of ischemic stroke are used to study the basic pathophysiological processes and potential therapeutic interventions in this disease; the extension of knowledge gained from these animal models will lead to improvement of medical treatment for human ischemic stroke in the future. Focal cerebral ischemia by middle cerebral artery occlusion (MCAO) in rats or mice is the rodent model most immediately relevant to human stroke. Using this method, transient ischemia is achieved by inserting a suture into the left middle cerebral artery, temporarily blocking blood flow to the middle cerebral artery territory, and removing the suture to allow reperfusion after a duration of minutes to hours depending on the specific study [9, 19, 20].

 Glucose deprivation (GD) and combined oxygen-glucose deprivation (OGD) are common *in vitro* models of brain ischemia. Either cell cultures or slice cultures are subjected to medium lacking glucose, and in the case of OGD, also placed in a chamber with very low oxygen levels for a fixed period of time [19, 21–26], followed by restoration of oxygen and glucose to the medium to imitate reperfusion.

## 3. Molecular Chaperones

Molecular chaperones were originally defined as a functionally related group of proteins that assist protein folding in bacterial, plant, and animal cells. The heat shock proteins of the 70 kDa molecular weight family (HSP70), including HSP72 (cytosol), GRP75/mortalin (mitochondria), and GRP78/BIP (endoplasmic reticulum; ER), are highly evolutionarily conserved and have been extensively studied. Studies, including those from our laboratory, show that all three of these HSP70 family members are protective in both *in vivo* and *in vitro* models of stroke [19, 27–32]. It has been a long-standing observation, as documented for HSP72 [33–35] and GRP75 [36], that cells destined to die fail to produce heat shock proteins, while cells that survive make new heat shock proteins. We recently identified translational arrest of GRP78 due to microRNA181 in focal cerebral ischemia in the mouse [19]. Although *Grp78* mRNA was induced following MCAO both in the core and outside the infarcted area, GRP78 protein was only induced in the penumbra, not within the area of infarction.

Recently a more complex, integrating role of these proteins has been recognized, that of stabilizing intracellular morphological and functional networks through protein-protein interactions with numerous client proteins [37–39]. This chaperoning network concept is increasingly accepted as a basic regulatory mechanism in diverse cellular functions [39, 40]. These networks allow the cell to change phenotype by releasing client proteins from chaperones allowing them to be activated, or in some cases released and degraded. These functional adjustments are rapid, do not require protein synthesis, and facilitate calibrated and integrated adaptation to changing conditions.

In addition to the new concept of the chaperone network, each individual chaperone has been found to have additional functions beyond that of functioning as a molecular chaperone. For example, GRP78 is traditionally considered to be a major endoplasmic reticulum chaperone as well as a master regulator of the unfolded protein response. Due to recent findings that significant amounts of GRP78 are present on the surface of cancer cells, it has emerged as an important regulator of tumor cell viability signaling, and cell surface GRP78 is now being used for therapeutic targeting [41]. In addition to GRP78, the ER calcium-binding protein calreticulin has also been shown to traffic to the plasma membrane and be involved in regulation of cell death [42, 43]. GRP78 plays a critical role in physiologic and pathologic stress [44], including developmental and neurological disorders [45]. As a multifunctional receptor on the cell surface [46], GRP78 may be associated with the AKT and ERK signaling pathways [47]. Because of its multiple locations and functions, GRP78 may play a central role in the chaperone network. HSP72 also protects brain by regulating distinct pathways of apoptosis and inflammation which both contribute to outcome after cerebral ischemia (for review see [48]). Other ER proteins also participate in cell death regulation, and function outside the ER.

## 4. The Mitochondria-Associated ER Membrane (MAM)

Although the association of endoplasmic reticulum (ER) with mitochondria was first observed in the 1960s by several independent groups [49, 50], morphological evidence for the physical association or interaction between the ER and mitochondria first emerged in the early 1990s. Such contact has since been observed in mitochondria in many types of cell [51, 52]. Structural and functional interactions of mitochondria with the ER have been demonstrated for rat brain [53]. The close contacts through which ER communicates with mitochondria are referred to as MAM [54]. The distance between the ER and the outer mitochondrial membrane (OMM) was originally estimated to be approximately 100 nm [51, 52]. However, a more recent study using electron tomography showed that the minimum distance is even less, 10 nm at the smooth ER and 25 nm at the rough ER [55]. Actually the spacing between the ER and mitochondria changes with different cell physiological and pathological conditions [56, 57] and artificial modification of this contact can lead to ER stress [55].

Numerous proteins have recently been proposed to participate in the interaction and communication between the mitochondria and the ER, highlighting the emerging role of this region in bioenergetics, cell survival, and cell death [58, 59]. One important structure is the IP3R on the ER and the voltage-dependent anion channel (VDAC) on the OMM which are now thought to be physically coupled through the chaperone Grp75/mortalin (Figure 1(b)) [60]. The sigma-1 receptor (SIG1R) chaperone is enriched in the MAM fraction [61–64] and recruits GRP78. In addition, other Ca^2+^-binding ER resident chaperones have been found in the MAM fraction, for example, calnexin (CNX), calreticulin, and ERp44 [65–67]. The multifunctional cytosolic sorting protein PACS-2 is another protein that has been found in the MAM fraction [68]. This fraction can also contain adenine nucleotide translocase (ANT) and cyclophylin D, the components of mitochondrial contact sites with similar composition to the mitochondrial permeability transition pore (MPTP). Such close apposition of the MPTP to the ER can sensitize mitochondria to Ca^2+^ signals [69]. Recently, the mitochondrial GTPase mitofusin 2 has been shown to be enriched in MAM as well as localized on the ER, where it interacts with mitofusins on mitochondria to form interorganellar bridges [70].

MAM can be isolated from tissues and cells to investigate the mechanisms and functions involved [60, 71]. Wieckowski et al. provided detailed protocols in 2009 in Nature Protocols [71]. Briefly the procedure consists of two steps: a crude mitochondrial fraction is isolated from tissue or cells by differential centrifugation, and the crude mitochondria are fractionated to the pure mitochondria and MAM fraction by Percoll density gradient.

## 5. Ca^2+^ Signaling at the MAM during Apoptosis

It is commonly accepted that the main structure responsible for ER-mitochondrial calcium transfer at the MAM is composed of the IP3R on the ER, VDAC on the OMM and MCU on the IMM (Figure 1(b)). Ca^2+^ released upon activation of the IP3R at the ER is taken up into mitochondria via VDAC and then MCU [72, 73].

A major function of MAM is the control of Ca^2+^ signaling between ER and mitochondria, a central topic of major interest both in normal physiology and pathophysiology. This second messenger has been proposed to have multiple roles in modulating intracellular events including bioenergetics and autophagy. Constitutive calcium release via the IP3R was found to be essential for maintaining normal bioenergetics and suppressing autophagy in conditions of ready nutrient availability [74]. In contrast during ER stress, Ca^2+^ increase seems to be required for triggering autophagy [75], though calcium-independent routes to induce autophagy involving interaction of IP3R with Beclin have also been reported [76], and lack of Ca^2+^ release via the IP3R can also induce autophagy [74]. Thus the role of calcium is complex, and induction of autophagy reflects combined input from Ca^2+^ dependent and independent pathways (see recent review [77]). Under prolonged ER stress conditions, as happens in the ischemic core after cerebral ischemia, a slow but sustained increase in mitochondrial matrix free [Ca^2+^] can occur, which can reach a critical threshold to trigger the opening of MPTP and initiate the apoptotic cascade (Figure 1(a)). Some studies indicate that the induction of apoptosis by ER stress has a mandatory mitochondrial component, further highlighting the intimate connection between these two organelles [78].

The ER can play an important role in regulating apoptosis by adjusting the load of Ca^2+^ imposed upon the mitochondrion. Previous studies have shown that the reduction in the Ca^2+^ amount that can be released from ER to mitochondria decreases the probability of Ca^2+^-dependent apoptosis. On the other hand, conditions that increase ER Ca^2+^ storage have the opposite effect on Ca^2+^-dependent apoptosis [79–82]. It has been demonstrated that overexpression of the antiapoptotic protein BCL2 can influence the distribution of Ca^2+^ within the ER/mitochondrial complex. Knockout of the proapoptotic proteins BAX and BAK reduced the resting concentration of ER Ca^2+^ decreasing the uptake of Ca^2+^ by mitochondria after Ca^2+^ release from the ER [81]. The active form of the antiapoptotic protein AKT results in reduced ER Ca^2+^ release, and diminished cellular sensitivity to Ca^2+^-mediated apoptotic stimuli [79, 82]. Antiapoptotic proteins BCL2 and AKT affect ER calcium homeostasis by differential mechanisms: BCL2 overexpression increases the Ca^2+^ leak from the ER, while AKT hyperactivation induces a decrease in ER Ca^2+^ release, probably through phosphorylation of the IP3R [58, 80].

## 6. Molecular Chaperones Regulate MAM

Some important chaperones are enriched in MAM and may play a key role in regulating Ca^2+^ signaling between ER and mitochondria. It was found that the mitochondrial chaperone GRP75 regulates IP3R-mediated mitochondrial Ca^2+^ signaling [60]. It was demonstrated that isoform 1 of VDAC is physically linked to the ER Ca^2+^-release channel IP3R through GRP75, highlighting chaperone-mediated conformational coupling between the IP3R and the mitochondrial Ca^2+^ uptake machinery (Figure 1(b)). We have found that overexpression of GRP75 improved mitochondrial function after *in vivo* and *in vitro* cerebral ischemia [31, 83].

ER protein SIG1R, implicated in neuroprotection, carcinogenesis, and neuroplasticity, is a Ca^2+^-sensitive and ligand-operated receptor chaperone at the MAM [62]. Normally, SIG1R forms a complex at the MAM with another ER chaperone GRP78/BiP (Figure 1(b)). Upon ER Ca^2+^ depletion or after ligand stimulation, SIG1R can dissociate from GRP78 and begin to chaperone conformationally unstable IP3R (Figure 1(b)) to enhance Ca^2+^ signaling from the ER into mitochondria to increase the production of ATP in the cell through the tricarboxylic acid cycle in the mitochondria [74]. If stimulated by high concentrations of agonists or impacted by extreme ER stress, SIG1Rs translocate from the MAM to the plasma membrane to bind various ion channels, receptors, or kinases [63, 84–86]. An increase of SIG1R in cells counteracts ER stress, whereas decreased levels enhance apoptosis.

**Figure 2:**
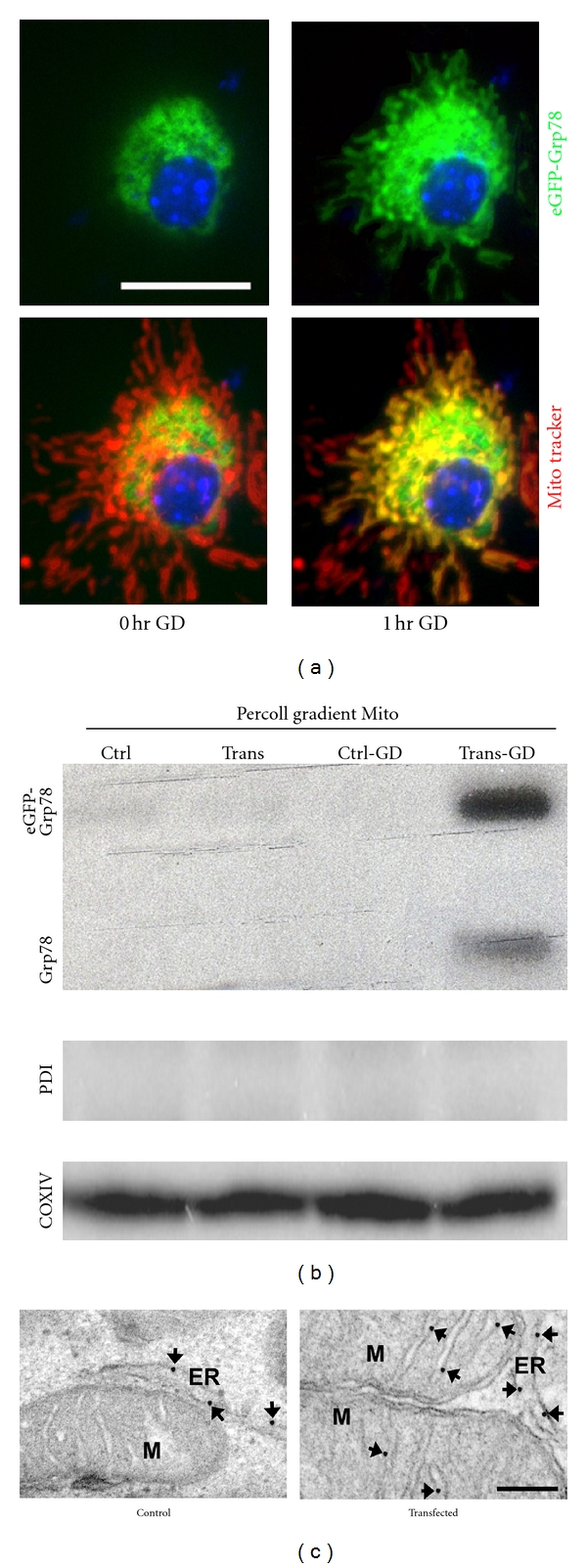
GRP78 retargets to mitochondria with glucose deprivation (GD). (a) Fluorescence photomicrographs were taken before and after 1 hr GD. Under normal conditions, the green fluorescence in eGFP-Grp78 transfected cells shows the normal perinuclear ER localization. After 1 hr GD this changes to a diffuse cytoplasmic pattern overlapping with the mitochondrial distribution visualized by partial overlap with Mito-tracker (red) fluorescence. Overlap is yellow. (b) After 3 hr GD, mitochondria were purified and analyzed by Western blotting using antibodies against GRP78, PDI (an ER-specific marker), or COXIV (a mitochondrial marker). The purified mitochondria do not show contamination with ER marker PDI but do have GRP78. Ctrl: control. Trans: transfected. (c) Submitochondrial localization of GRP78 in control and GD-stressed cells by immunoelectron microscopy. Arrows point to the localization of GRP78. The gold particles were associated exclusively with ER membrane in control cells (left panel). In contrast, immune-EM staining shows GRP78 within mitochondria from 3 hr GD-stressed cells, demonstrating significant mitochondrial labeling, with grains mainly decorating the inner mitochondrial membrane (right panel). M, mitochondrion. Scale bars, 100 nm.

Recent evidence [41] indicates that GRP78, like SIG1R, may emerge as a novel interorganelle signaling modulator. As a multifunctional receptor on the cell surface after stress [46], GRP78 may be associated with many signaling pathways [47]. However, until now there has been no detailed research on the importance of GRP78 in MAM except as a binding partner of SIG1R (Figure 1(b)). GRP78 has been found to be one of the VDAC interactors (Table  1 in [60]) together with GRP75, although the authors of the paper never discuss it in the text [60]. We recently found that overexpressing GRP78 preserves respiratory activity and mitochondrial membrane potential, reduces free radical production, reduces mitochondria Ca^2+^ overload, and increases Ca^2+^ uptake capacity in isolated mitochondria after stress [22]. In order to follow GRP78 directly in response to ischemia-like stress, we created a fusion protein consisting of green fluorescent protein (eGFP) fused between the GRP78 N-terminal 18 amino acid ER signal peptide and the remainder of GRP78. We found that eGFP-GRP78 retargets to mitochondria within a short period of GD by fluorescence and immunoelectron microscopy (IEM) as well as Western blotting (Figure 2). The mitochondrial location of GRP78 is mainly on the inner membrane of mitochondria by IEM (Figure 2(c)). A prior report in 9L tumor cells has demonstrated relocalization of GRP78 to mitochondria after induction of ER stress by thapsigargin [87]. As in the case of translocation to the cell surface, cytoplasm, and nucleus after stress [41], the molecular mechanism underlying GRP78 translocation to mitochondria has not yet been elucidated.

The mitochondrial Ca^2+^ uniporter is the primary influx pathway for Ca^2+^ into respiring mitochondria, and hence is a key regulator of mitochondrial Ca^2+^. Although the uniporter's biophysical properties have been studied extensively, its molecular composition remained elusive for more than 50 years. A very recent report has identified a 40-kDa protein which fulfills the requirements for being the long sought mitochondrial calcium uniporter [72]. Overexpression of MCU alone in one report did not give rise to a marked gain of Ca^2+^ uptake in HeLa cells indicating that additional components or chaperones may be limiting in some settings [88], though other investigators did observe increased Ca^2+^ with overexpression [72]. The MCU is thought to function as part of a complex including at least MICU1 [89]. Considering the fact that overexpressing GRP78 not only reduces mitochondria Ca^2+^ overload in intact cells, but also increases Ca^2+^ uptake capacity in isolated mitochondria [22], it is possible that translocated GRP78 interacts with the uniporter in some way on the IMM and regulates the mitochondrial Ca^2+^. Future validation of the hypothesis depends on further development of molecular approaches to confirm this property of MCU and its relationship with GRP78.

In summary these findings together support a new emerging picture: chaperone machineries at both the ER and mitochondrion orchestrate the regulation of Ca^2+^ signaling between these two organelles and control bioenergetics, cell survival, and cell death decisions. In the brain, ER calcium release has been found to directly contribute to excitotoxicity, a neuronal death mechanism important both in acute and chronic neurodegenerative diseases. Better understanding the roles of chaperones and Ca^2+^ handling *in vivo *should in the future provide new therapeutic strategies to protect brain cells during ischemia.
